# Ag/AgCl Nanoparticle Incorporation into *Epipremnum aureum* for Electrothermal Signal Amplification and Machine-Learning-Based Temperature Prediction

**DOI:** 10.3390/bios16080450

**Published:** 2026-08-19

**Authors:** Marco Merino-Treviño, Ana Beatriz Morales-Cepeda, Hernán Peraza-Vázquez, Edgar Onofre-Bustamante

**Affiliations:** 1 Tecnológico Nacional de México (TecNM), Instituto Tecnológico de Ciudad Madero, Ciudad Madero 89440, Tamaulipas, Mexico; mmerino@ipn.mx; 2 Instituto Politécnico Nacional, Centro de Investigación en Ciencia Aplicada y Tecnología Avanzada (CICATA), Unidad Altamira, Altamira 89600, Tamaulipas, Mexico; eonofre@ipn.mx

**Keywords:** plant biosensor, *Epipremnum aureum*, Ag/AgCl nanostructures, plant nanobionics, electrothermal transduction, temperature sensing, bioelectrical signals, machine learning, temperature prediction, precision agriculture

## Abstract

Recently, plant-based bioelectronic systems have been explored for environmental sensing applications. However, their intrinsically low electrical conductivity often limits signal sensitivity and measurement reliability. In this work, the electrothermal behavior of living *Epipremnum aureum* plants incorporating Ag/AgCl nanoparticles supported on nanocellulose was investigated. Electrical and thermal responses were simultaneously measured under controlled environmental conditions using external shunt resistances of 1, 10, 100, and 1000 Ω. Compared with the control without nanoparticle incorporation, the nanoparticle-incorporated plant exhibited stronger electrical responses and distinct electrothermal behavior over the studied temperature range. The measured signals showed nonlinear responses, temporal asymmetry, and resistance-dependent modulation, suggesting changes in charge transport within the plant tissues. Silver-enriched regions and the co-detection of chlorine within the nanoparticle-incorporated plant tissues were identified by environmental scanning electron microscopy and energy-dispersive X-ray spectroscopy. Five machine-learning regression models were trained to estimate temperature using the measured electrothermal voltage signals as predictors. The best-performing model, MLP FitRNet, achieved a mean absolute error of 0.598 °C, a root mean square error of 0.748 °C, and an $R^{2}$ value of 0.974. These results demonstrate the potential of nanoparticle-incorporated biohybrid plant systems for electrothermal signal analysis and data-driven temperature estimation, while providing a foundation for future intelligent environmental monitoring applications.

## 1. Introduction

The rising demand for sustainable and efficient agricultural systems has increased the need for advanced tools to monitor plant physiological responses in real time. Environmental stresses such as temperature variations, drought, salinity, and soil variability can substantially affect plant growth, metabolism, and productivity. However, detecting these responses at an early stage remains challenging because conventional approaches, including imaging, spectroscopy, and remote sensing, generally provide indirect or macroscopic information. Consequently, there is growing interest in sensing strategies capable of directly capturing physiological processes in living plants.

Plants are dynamic biological systems that continuously interact with their environment. As sessile organisms, they have evolved signaling networks that allow them to perceive and respond to external stimuli. Electrical signaling is a fundamental mechanism for long-distance communication in plant tissues and originates from ion fluxes across cellular membranes, ion-channel activity, hydraulic interactions, and electrophysiological propagation through vascular tissues [1,2,3,4,5]. These signals participate in physiological processes including stress responses, nutrient transport, photosynthesis, respiration, phytohormone synthesis, and environmental adaptation [1,4,6].

Environmental factors such as light, mechanical stimulation, water availability, and temperature can induce measurable changes in plant electrical activity by affecting ion mobility, membrane permeability, hydraulic redistribution, and metabolic processes. Plant bioelectrical signals can therefore provide information about both physiological state and environmental conditions. Recent studies have demonstrated that these signals can be recorded under environmental conditions and interpreted using data-driven approaches [7,8]. Thermal and light stimulation can also induce propagating electrical responses associated with systemic physiological processes [9,10]. Nevertheless, native plant electrical signals generally exhibit low amplitude, substantial biological variability, and susceptibility to environmental noise, which can limit their direct use as reliable sensing outputs [4,5,6].

Plant nanobionics offers a potential strategy for modifying these responses through the incorporation of engineered nanomaterials into living plants [11,12,13,14,15]. Conductive nanostructures can interact with biological charge-transport processes and may modify measurable electrical responses, providing opportunities to investigate biohybrid plant–nanomaterial systems. Nanobiosensor-based approaches have consequently attracted interest in agricultural applications because nanomaterials may contribute to signal amplification, sensitivity, and in situ monitoring [16].

Among conductive nanomaterials, silver-based nanostructures are of particular interest because of their electrical and electrochemical properties. Previous studies have shown that silver nanoparticles can interact with plant roots and induce physiological and molecular responses in plant tissues [17,18]. Their uptake, chemical transformation, and distribution in plants depend on factors such as nanoparticle composition, surface properties, concentration, and plant species [18]. More broadly, experimental studies have demonstrated that engineered nanoparticles can penetrate plant roots and subsequently move from roots to shoots, supporting the feasibility of nanoparticle transport through plant tissues [19]. Within this research context, Ag/AgCl nanostructures supported on nanocellulose provide a dispersible platform suitable for investigating interactions between conductive nanostructures and living plant tissues [20]. In our previous work [21], silver nanoparticles supported on nanocellulose were incorporated into *Epipremnum aureum*, and structural characterization provided evidence of their presence within plant tissues. Electrical measurements also showed changes in signal response compared with plants without nanoparticle incorporation. These observations motivated further investigation of the electrothermal behavior associated with Ag/AgCl incorporation.

Despite advances in plant nanobionics and electrophysiology, previous studies have mainly focused either on modifying plant functions using nanomaterials [11,13,14] or on characterizing intrinsic plant electrical signaling [1,2,4,5]. However, the relationship between nanomaterial incorporation and electrothermal signal transduction in living plants remains insufficiently understood. In particular, the nonlinear and non-ohmic electrical behavior associated with Ag/AgCl incorporation and its response to thermal stimulation have not been systematically characterized.

Machine-learning methods provide an additional tool for investigating these complex relationships because they can identify nonlinear patterns between bioelectrical responses and environmental variables that may be difficult to describe using conventional analytical models. In this study, machine-learning regression was therefore used to evaluate whether measured electrothermal voltage signals contain sufficient information for data-driven temperature estimation.

Accordingly, this work investigates the electrothermal behavior of *Epipremnum aureum* after the incorporation of Ag/AgCl nanostructures supported on nanocellulose. The specific objectives are: (i) to quantify changes in electrical signal magnitude associated with nanoparticle incorporation; (ii) to analyze the relationship between temperature and electrical response under different external resistive loads; (iii) to provide structural and compositional evidence of Ag/AgCl within plant tissues; and (iv) to evaluate the feasibility of estimating temperature from electrothermal voltage signals using machine-learning regression models.

## 2. Materials and Methods

### 2.1. Experimental System Overview

**Figure 1:**
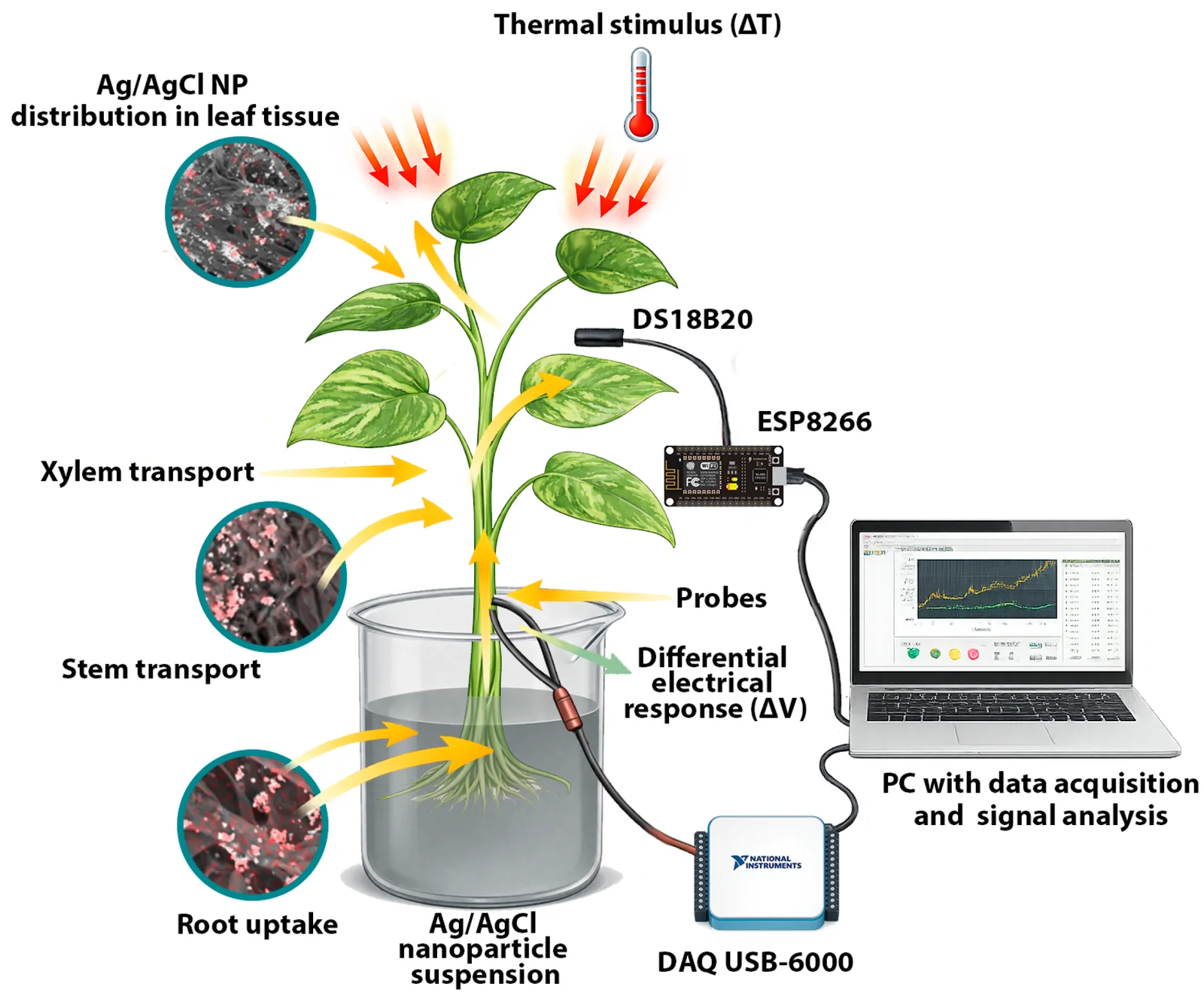
Conceptual schematic of the proposed biohybrid sensing system based on Ag/AgCl nanoparticle-incorporated *Epipremnum aureum*. The experimental workflow includes nanoparticle incorporation, electrothermal signal acquisition under controlled resistive loads, data acquisition and processing, and machine-learning-based temperature estimation.

The experimental biohybrid sensing system is schematically illustrated in Figure 1. The system integrates Ag/AgCl nanoparticle-incorporated *Epipremnum aureum*, external stainless-steel electrodes, controlled resistive loads, simultaneous voltage and temperature acquisition, and machine-learning-based data analysis. Ag/AgCl nanostructures supported on nanocellulose were introduced into the plant and allowed to undergo passive uptake during the exposure period. Electrical responses were subsequently measured using external electrodes while plant temperature was monitored simultaneously. The acquired electrothermal voltage signals were processed and used as predictor variables for data-driven temperature estimation. Figure 1 summarizes the experimental workflow from nanoparticle incorporation and electrothermal signal acquisition to computational analysis.

### 2.2. Chemicals and Reagents

The α-cellulose powder (average particle size of 50 μm) and 2,2,6,6-tetramethylpiperidine-1-oxyl (TEMPO) were obtained from Sigma-Aldrich (St. Louis, MO, USA). Sodium bromide (NaBr), silver nitrate (AgNO_3_), sodium hypochlorite (NaOCl, 6–9%), sodium hydroxide (NaOH), and ethanol were obtained from Fermont (Productos Químicos Monterrey, Monterrey, Nuevo León, Mexico). Deionized water was used for suspension preparation, dilution, and washing. All chemical reagents were used as received without further purification.

### 2.3. Synthesis and Preparation of the Ag/AgCl Nanostructure Suspension

**Figure 2:**
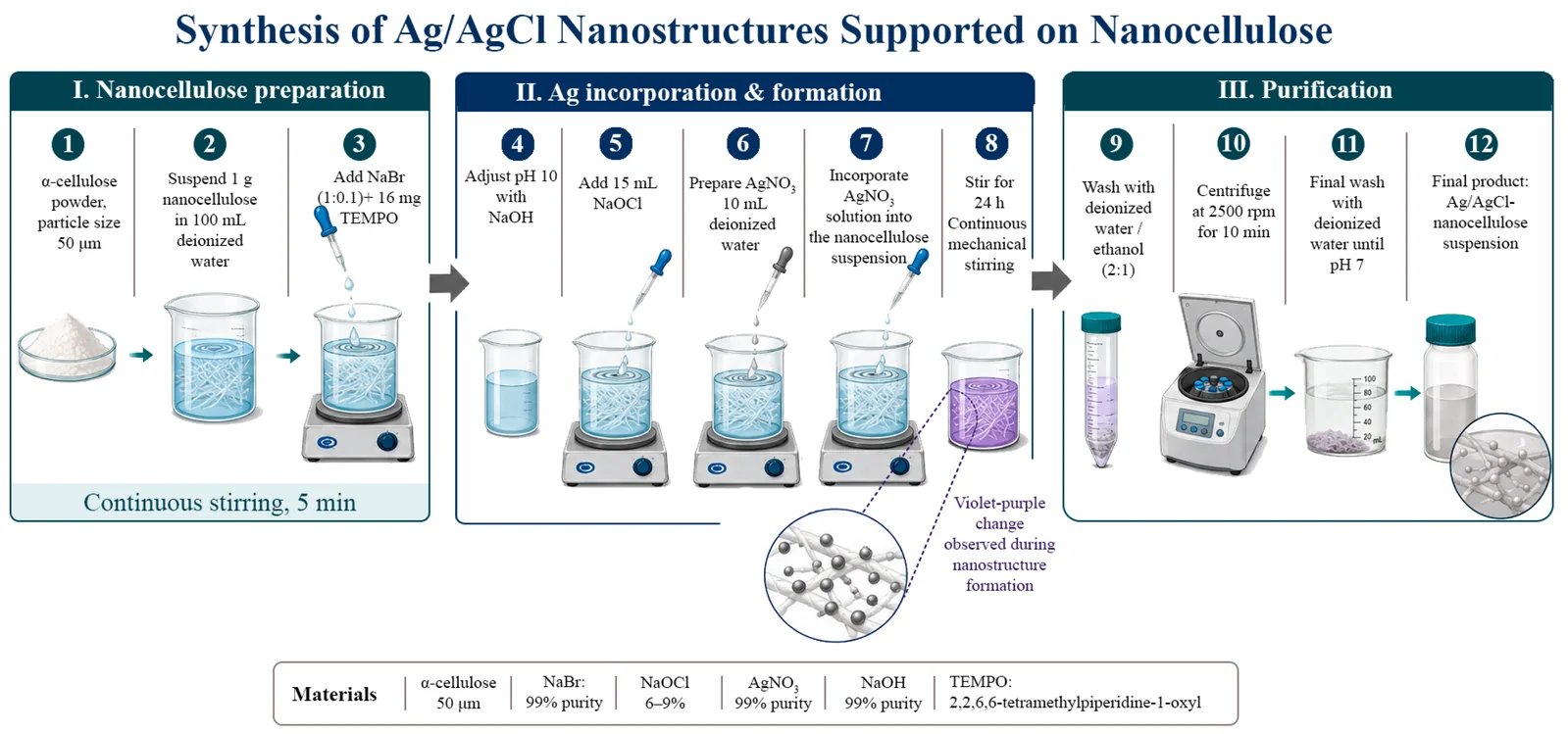
Schematic representation of the one-step in situ synthesis of Ag/AgCl nanostructures supported on nanocellulose, including the main reaction, purification, and suspension-preparation steps. The procedure was based on Macclesh del Pino Pérez et al. [20] and our previously reported methodology [21].

Ag/AgCl nanostructures supported on nanocellulose were prepared using a one-step in situ synthesis based on the methodology reported by Macclesh del Pino Pérez et al. [20] and previously employed in our work [21]. The complete synthesis procedure is schematically summarized in Figure 2.

Briefly, 1 g of α-cellulose (average particle size of 50 μm) was suspended in 100 mL of deionized water. Sodium bromide (NaBr) was then added at a cellulose:NaBr mass ratio of 1:0.1, followed by 16 mg of 2,2,6,6-tetramethylpiperidine-1-oxyl (TEMPO). The suspension was maintained under continuous mechanical stirring.

Silver nitrate (AgNO_3_) was separately dissolved in 10 mL of deionized water and subsequently incorporated into the α-cellulose suspension. The mixture was mechanically stirred for 5 min. Subsequently, 15 mL of sodium hypochlorite (NaOCl) was added, and the pH was adjusted to 10 by the dropwise addition of NaOH. The pH was monitored throughout the reaction, and NaOH was added as required to maintain the alkaline conditions.

After stabilization at pH 10, the suspension was maintained under continuous mechanical stirring for 24 h at room temperature. During this period, a progressive violet–purple color change was observed during the formation of the Ag/AgCl-containing nanostructures.

The resulting suspension was washed with a 2:1 mixture of deionized water and ethanol and centrifuged at 2500 rpm for 10 min. Additional washing with deionized water was performed until the suspension reached pH 7. The resulting Ag/AgCl nanostructures supported on nanocellulose were maintained as an aqueous suspension for subsequent incorporation into the plant.

For the experiments described in this study, the Ag/AgCl-containing aqueous suspension was adjusted to 10 wt% and homogenized by mechanical stirring before plant application. Neither ultrasonication nor vortex mixing was used to redisperse or homogenize the final suspension.

### 2.4. Plant Material and Nanostructure Incorporation

The biological model was chosen as healthy specimens of *Epipremnum aureum* due to their strong vascular architecture, high environmental adaptability, and previously reported ability to uptake and transport nanoparticles. Plants were acclimated to controlled environmental conditions prior to experimentation to ensure stable physiological responses and minimize variability due to external stress.

Nanoparticle incorporation into the plant was performed using an aqueous suspension of Ag/AgCl nanostructures supported on nanocellulose at a concentration of 10 wt%. A volume of 5 mL of the suspension was applied locally to the root region of the nanoparticle-incorporated specimen, as illustrated in Figure 1, allowing passive uptake through the roots and subsequent transport through the vascular system. This procedure avoided direct injection of the suspension and additional invasive manipulation of the vascular tissues during nanoparticle administration.

The plants were maintained in continuous contact with the nanomaterial suspension for 12 weeks to allow gradual nanoparticle uptake and distribution within the plant tissues. After the exposure period, the plants were subjected to a 48 h stabilization period before electrical characterization to minimize transient physiological effects associated with handling and nanoparticle administration.

### 2.5. Electrical Measurement Setup

Electrical signals were acquired using a DAQ USB-6000 system (National Instruments, Austin, TX, USA) for high-resolution voltage measurements. The acquisition system was interfaced with a microcontroller platform (ESP8266) for data logging and synchronization.

**Figure 3:**
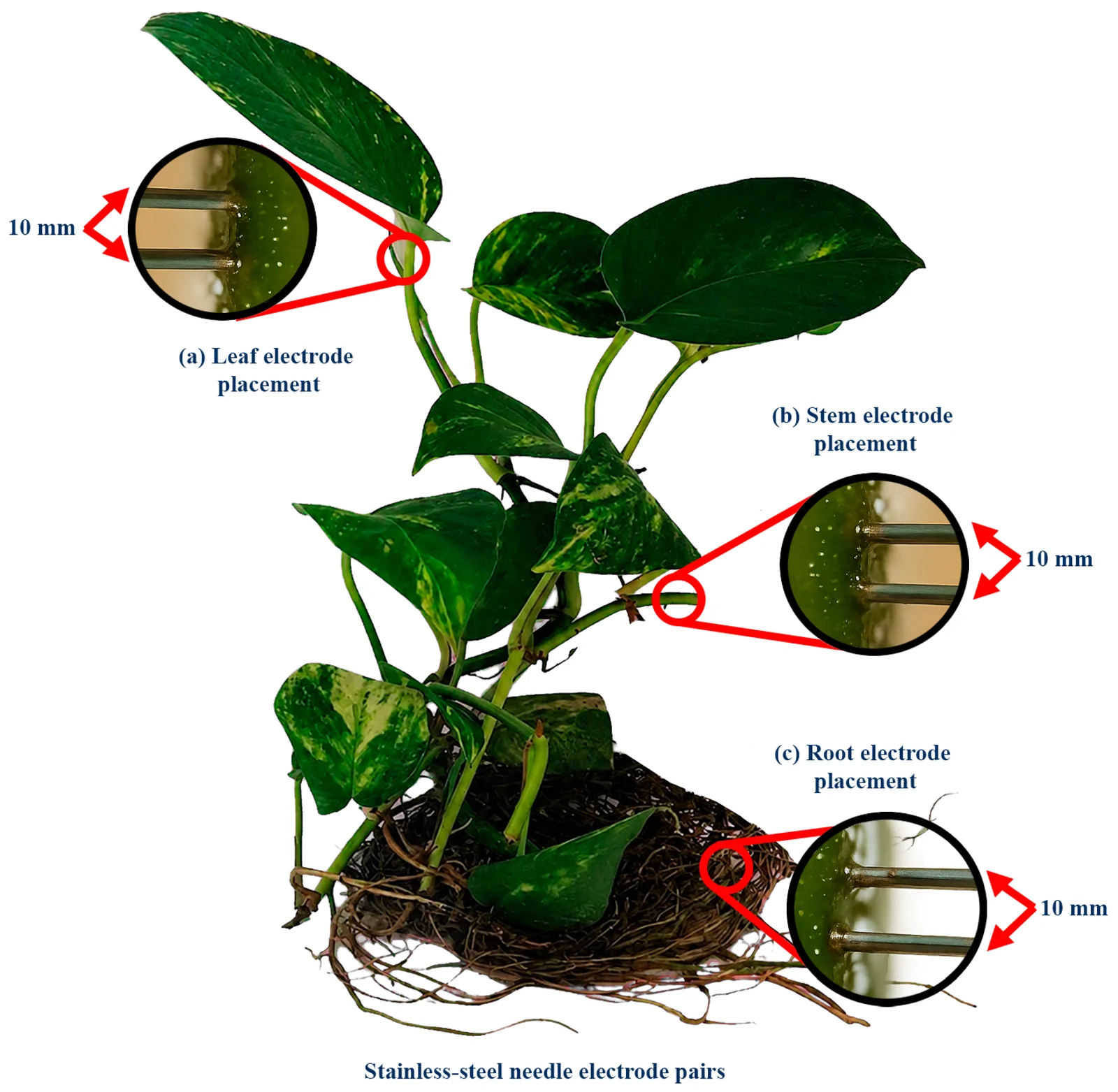
Schematic representation of the voltage-measurement configuration and electrode placement in *Epipremnum aureum*: (**a**) leaf, (**b**) stem, and (**c**) root. The enlarged views show pairs of stainless-steel needle electrodes (diameter ∼0.5 mm) inserted into the corresponding plant tissues with a fixed electrode-to-electrode separation of 10 mm. Red circles and connecting lines indicate the electrode insertion sites and their enlarged views.

Differential electrical signals were recorded by placing stainless-steel needle electrodes (diameter ∼0.5 mm) at specific locations corresponding to the leaf, stem, and root. Stable electrical contact with the plant tissues was maintained throughout the measurements. The electrode-placement configuration used for the electrothermal characterization is shown in Figure 3.

As illustrated in Figure 3, the voltage response was measured using pairs of stainless-steel needle electrodes inserted into the leaf, stem, and root tissues, with a fixed electrode-to-electrode separation of 10 mm. The electrodes were connected to the NI USB-6000 data-acquisition system (Austin, TX, USA), and the voltage response was recorded under the selected external resistive load. The electrode placement and separation were kept constant for each tissue throughout the measurements to minimize variations associated with the electrode geometry.

To characterize the electrical response under varying circuit conditions, external resistive loads of 1, 10, 100, and 1000 Ω were connected sequentially. Each resistance condition was maintained for 5 min, during which voltage readings were continuously recorded.

Measurements were performed using one Ag/AgCl nanoparticle-incorporated *Epipremnum aureum* specimen and one control specimen of the same species without nanoparticle incorporation. Accordingly, the experimental design was established as a proof-of-concept study to evaluate the feasibility of nanoparticle-assisted electrothermal sensing under the experimental conditions considered.

### 2.6. Temperature Monitoring

Temperature was monitored using a DS18B20 digital sensor placed in direct contact with the stem surface at the same internode level where electrical measurements were performed. The sensor was positioned adjacent (∼1 cm) to the electrode insertion point to ensure spatial correlation between thermal and electrical signals.

Temperature data were recorded simultaneously with electrical measurements using a shared timestamp to ensure temporal synchronization. Measurements covered nominal temperatures from 25 to 41 °C, corresponding to 17 temperature levels.

### 2.7. Data Acquisition and Processing

Signals were acquired at a sampling frequency of 1 Hz and transmitted to a processing unit for storage and analysis. Raw voltage signals were processed using a Savitzky–Golay filter with a second-order polynomial and a window size of 51 points to reduce high-frequency noise while preserving the relevant signal characteristics.

The voltage enhancement factor (VEF) was calculated as the ratio between the complete voltage range of the Ag/AgCl NP-incorporated condition and that of the control without nanoparticle incorporation. For each condition, the voltage range was defined as $V_{\max}-V_{\min}$ over the complete analyzed interval. Therefore, the value used for the control without nanoparticle incorporation was $V_{\max,\mathrm{control}}-V_{\min,\mathrm{control}}$, even when its signal did not exhibit distinct local peaks. The same analysis interval and calculation procedure were applied to both conditions.

The electrothermal coefficient (dV/dT) was estimated for the Ag/AgCl NP-incorporated condition by applying a first-order linear regression to all temperature–voltage pairs within the complete analyzed interval, including observations recorded during both the increasing- and decreasing-temperature stages. The voltage–temperature relationship was expressed as V=aT+b, where the fitted slope *a* was reported as dV/dT. Therefore, the calculated coefficient represents the global electrothermal trend of the complete analyzed dataset rather than the local slope around a voltage minimum.

Measurement repeatability was evaluated in the leaf, stem, and root regions under the external-resistance conditions considered in this study. Repeated voltage acquisitions were obtained at fixed temperatures for each tissue region. For concise visualization, the stem region under an external resistance of 1 kΩ was selected as a representative case. Two hundred repeated acquisitions were analyzed at nominal temperatures of 25, 30, 35, and 40 °C, with each acquisition comprising 200 sequential voltage samples recorded at 1 Hz. At each sampling position, the mean voltage and standard deviation were calculated across the 200 acquisitions. The measured temperature was also expressed as mean ± standard deviation for each nominal temperature.

### 2.8. Machine-Learning-Based Temperature Prediction

To evaluate whether the electrothermal voltage response could be used for computational temperature estimation, a machine-learning regression analysis was performed using the experimental dataset obtained from the plant-based sensing system. Each data file contained the sample index, temperature, voltage response of the control condition without nanoparticle incorporation, and voltage response of the Ag/AgCl NP-incorporated condition. The temperature value was used as the regression target, whereas the voltage signals were used as predictor variables.

The voltage responses of the control plant and the Ag/AgCl NP-incorporated plant were acquired as separate experimental data streams. Observations corresponding to the same sampling instant and temperature level were temporally aligned and subsequently combined into a single predictor vector. Consequently, each observation contained two synchronized predictor variables: the voltage measured in the control condition without nanoparticle incorporation and the voltage measured in the nanoparticle-incorporated condition. The model was therefore designed to estimate temperature from the joint predictor space formed by both experimental conditions and not from either plant condition considered independently.

Table 1 summarizes the variables used in the machine-learning regression analysis.

The complete dataset comprised 340 acquisition files and 68,000 observations distributed across 17 nominal temperature levels. To reduce potential information leakage associated with observations belonging to the same acquisition sequence, data partitioning was performed at the acquisition-file level rather than at the individual-observation level.

The dataset was divided into 70% for model training, 15% for validation, and 15% for independent testing. This partition resulted in 238 acquisition files (47,600 observations) for training, 51 acquisition files (10,200 observations) for validation, and 51 acquisition files (10,200 observations) for testing, as summarized in Table 2.

The validation subset was used for hyperparameter selection. After hyperparameter selection, the final models were retrained using the combined training and validation subsets, corresponding to 85% of the acquisition files. The independent testing subset, comprising the remaining 15%, was reserved exclusively for final performance evaluation.

The predictor variables consisted exclusively of voltage signals acquired from the electrothermal experiments, whereas temperature was used only as the regression target variable. To reduce scale-dependent effects and improve model convergence, the predictor variables were normalized prior to training.

Five supervised regression algorithms were evaluated: linear regression, support vector regression (SVR) with a Gaussian kernel, random forest regression, gradient boosting regression, and a multilayer perceptron (MLP) neural network implemented using MATLAB R2025b FitRNet. These models were selected to represent linear, kernel-based, ensemble-learning, and neural-network approaches with different levels of complexity. All algorithms were evaluated using the same file-level data partition to allow a consistent comparison of predictive performance. Machine-learning approaches have previously been used to analyze plant electrophysiological signals for environmental sensing and physiological state assessment [7].

Model performance was evaluated using mean absolute error (MAE), root mean square error (RMSE), mean bias error (MBE), the coefficient of determination ($R^{2}$), and maximum absolute error (MaxAbsError). These metrics were used to quantify prediction accuracy, systematic bias, goodness of fit, and the maximum observed deviation between measured and predicted temperatures.

The machine-learning analysis was conducted to evaluate the feasibility of estimating temperature from electrothermal voltage signals under the experimental conditions represented in the dataset. The regression results provide proof-of-concept computational evidence of the temperature-estimation capability of the proposed approach.

### 2.9. Morphological and Compositional Characterization

The morphological characterization was performed using an environmental scanning electron microscope (ESEM, Carl Zeiss EVO LS10, Germany) under low-vacuum conditions (80 Pa) and an accelerating voltage of 30 kV. A backscattered electron detector was used to enhance compositional contrast within plant tissues.

Representative regions of the tissue structure were obtained by sectioning plant samples with a sterile scalpel, including leaves, stems, and roots. Samples were mounted on aluminum stubs using double-sided conductive carbon tape and examined without conductive coating to preserve the native tissue structure. ESEM images were acquired in greyscale and recorded in TIFF format with a resolution of 1024 × 768 pixels.

Elemental analysis was carried out using an energy-dispersive X-ray spectroscopy system (EDX, Bruker Quantax 200, Germany) attached to the ESEM. Measurements were performed under semiquantitative conditions. EDX mapping and localized analyses were conducted to assess the spatial distribution and presence of silver within plant tissues. Structural features associated with nanoparticle incorporation were visualized by ESEM imaging, whereas elemental confirmation of Ag-rich regions was obtained by EDX analysis. The simultaneous detection of Ag and Cl in the analyzed regions was considered consistent with the presence of Ag/AgCl-containing domains within the plant tissues.

## 3. Results

### 3.1. Representative Electrothermal Response Under Controlled Resistive Loads

**Figure 4:**
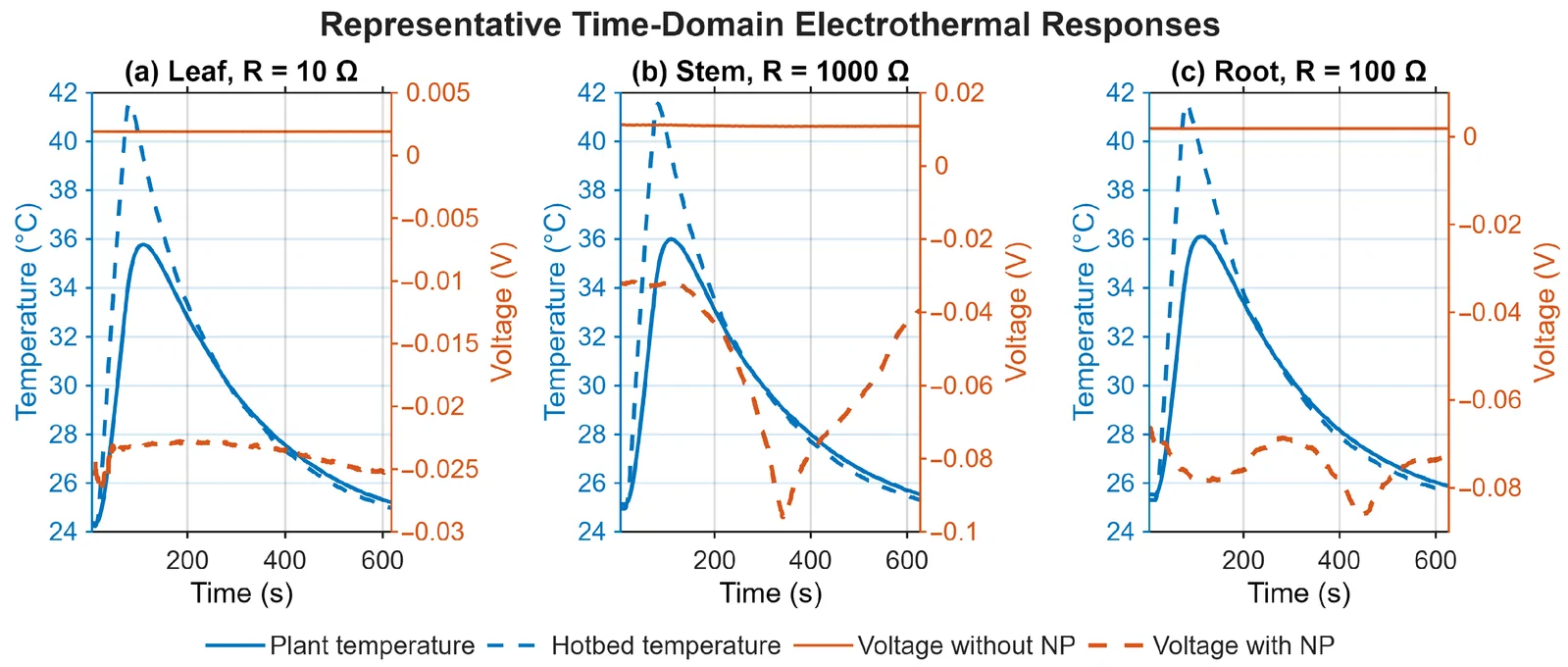
Representative time-domain electrothermal responses of *Epipremnum aureum* tissues under selected external resistance conditions: (**a**) leaf response at 10 Ω; (**b**) stem response at 1000 Ω; and (**c**) root response at 100 Ω. In all panels, the solid blue curve represents plant temperature, the dashed blue curve represents hotbed temperature, the solid orange curve represents the voltage response of the control condition without nanoparticle incorporation, and the dashed orange curve represents the voltage response of the Ag/AgCl NP-incorporated condition.

The electrical response of *Epipremnum aureum* was evaluated under controlled temperature variations and different external resistive loads. Representative time-domain responses for leaf, stem, and root tissues are shown in Figure 4. Differences in voltage amplitude and temporal response were observed between the control and Ag/AgCl NP-incorporated conditions.

Although the control signals appear nearly constant at the displayed scale, they were not completely noiseless. Small voltage variations were present throughout the analyzed intervals, as demonstrated by the nonzero voltage ranges ($V_{\max}-V_{\min}$) used in the VEF calculation and the nonzero RMSΔ*V* values obtained for the control condition without nanoparticle incorporation. However, their amplitudes were comparatively small and remained close to a steady baseline; consequently, they were not visually distinguishable at the voltage scale required to display the larger responses of the Ag/AgCl NP-incorporated plant. Thus, the apparently flat profiles should not be interpreted as an absolute absence of electrical variability, but rather as a comparatively weak response under the evaluated conditions.

Across the recorded signals, the electrical response exhibited nonlinear behavior, temporal asymmetry, and resistance-dependent modulation. The magnitude and polarity of the voltage response also varied according to plant tissue and external resistive load.

The thermal stimulus produced a rapid increase in hotbed temperature followed by a delayed and smoother variation in plant temperature. Simultaneously, the electrical signals exhibited changes in amplitude and temporal profile.

In the leaf region at 10 Ω (Figure 4a), the nanoparticle-incorporated plant showed a negative voltage response with a larger amplitude than the control condition without nanoparticle incorporation. In the stem region at 1000 Ω (Figure 4b), a pronounced transient negative excursion was observed during thermal relaxation. In the root region at 100 Ω (Figure 4c), the nanoparticle-incorporated condition exhibited a clear time-dependent voltage response throughout the thermal cycle. These representative responses were obtained under different external resistive loads; therefore, their voltage ranges were not used for direct quantitative comparison between tissues.

Overall, the representative measurements show that incorporation of Ag/AgCl nanoparticles was associated with amplified and tissue-dependent electrical responses under the evaluated electrothermal conditions.

### 3.2. Repeatability Under Fixed-Temperature Conditions

To assess measurement repeatability without excessively increasing the number of panels, the stem region was selected as a representative tissue. Two hundred repeated voltage acquisitions were analyzed at four fixed nominal temperatures (25, 30, 35, and 40 °C) using an external resistance of 1 kΩ. Each acquisition comprised 200 sequential samples.

**Figure 5:**
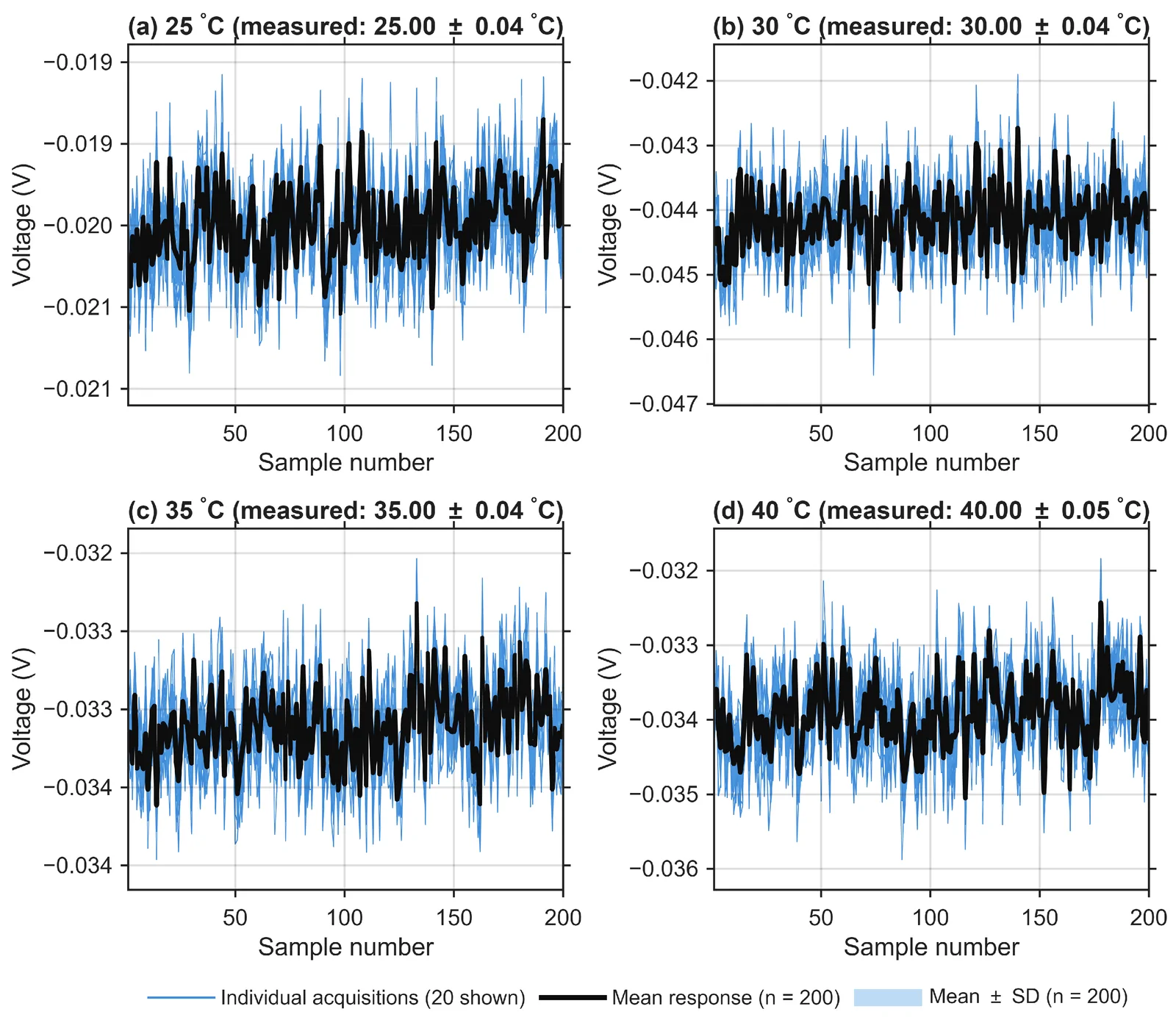
Repeatability of the voltage response measured in the stem region of the Ag/AgCl NP-incorporated *Epipremnum aureum* using an external resistance of 1 kΩ at nominal temperatures of (**a**) 25, (**b**) 30, (**c**) 35, and (**d**) 40 °C. Two hundred repeated acquisitions were analyzed at each temperature, with each acquisition comprising 200 sequential samples. The blue curves represent the individual acquisitions, the black curve represents the mean response calculated from the 200 acquisitions, and the shaded region indicates the mean ± standard deviation. The measured temperature is reported as mean ± standard deviation above each panel. The vertical scale was adjusted independently in each panel to facilitate visualization of within-temperature variability.

As shown in Figure 5, the repeated acquisitions were distributed around their corresponding mean responses. The measured temperatures remained stable at 25.00 ± 0.04, 30.00 ± 0.04, 35.00 ± 0.04, and 40.00 ± 0.05 °C. The clustering of the voltage responses and the corresponding mean ± standard-deviation bands supports the repeatability of the measurements in the stem region under fixed-temperature conditions.

### 3.3. Machine-Learning Prediction of Temperature from Electrothermal Signals

The feasibility of estimating temperature from the measured electrothermal voltage signals was evaluated using five supervised regression models. Table 3 summarizes the predictive performance obtained for the independent testing subset.

Among the evaluated models, MLP FitRNet achieved the lowest MAE (0.598 °C) and RMSE (0.748 °C), with an $R^{2}$ value of 0.974. Gradient Boosting exhibited similar performance, with an MAE of 0.601 °C, an RMSE of 0.752 °C, and an $R^{2}$ value of 0.974. The remaining models produced $R^{2}$ values ranging from 0.969 to 0.973.

**Figure 6:**
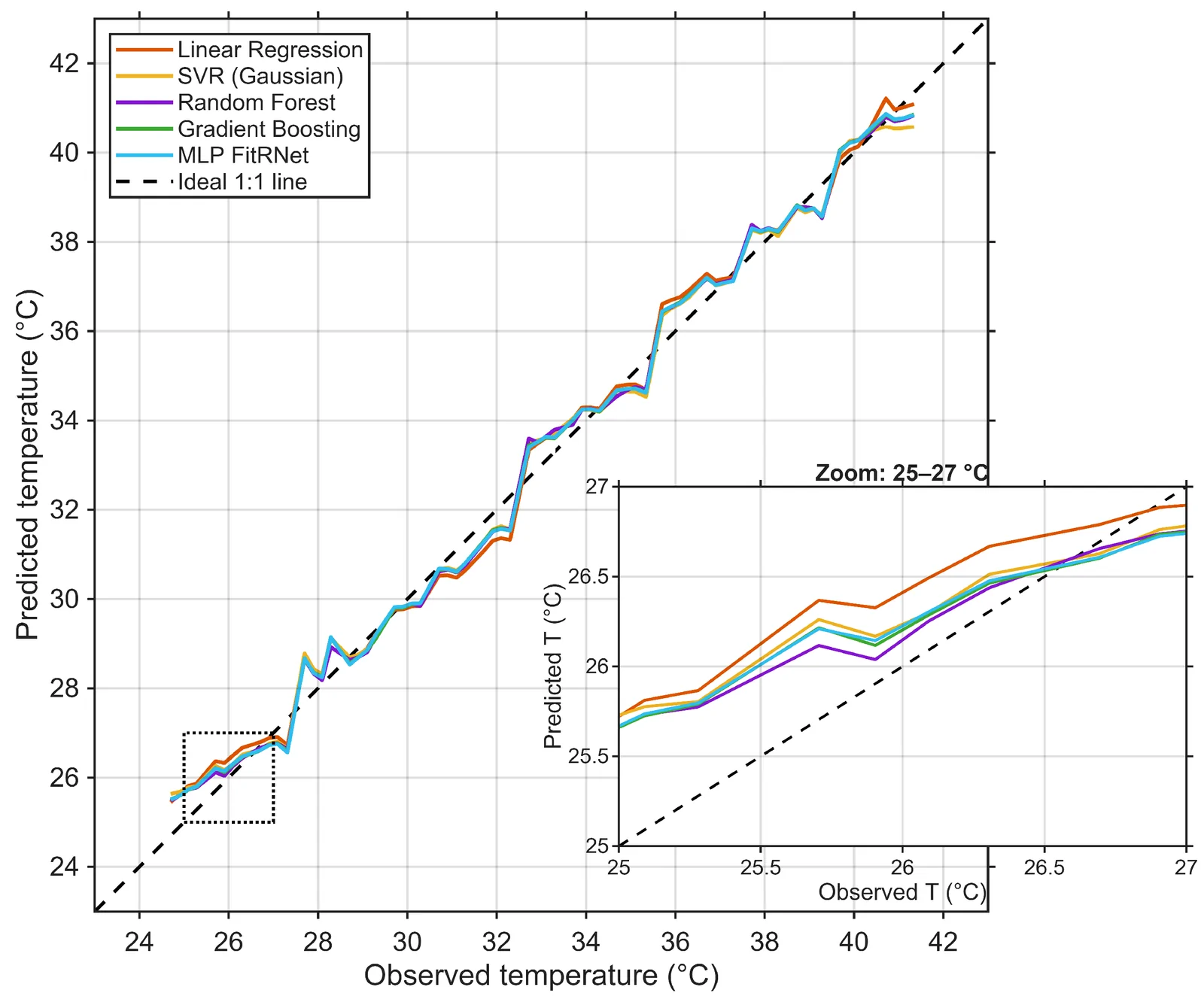
Measured versus predicted temperatures obtained using the five regression models evaluated on the independent testing subset (N=10,200 observations). The dashed line represents the ideal one-to-one relationship.

Figure 6 compares the measured and predicted temperatures obtained using the five regression models evaluated on the independent testing subset. Among the evaluated models, MLP FitRNet showed the closest agreement with the one-to-one reference line throughout the investigated temperature range. This model exhibited an MBE of 0.036 °C and a maximum absolute error of 2.77 °C.

### 3.4. Voltage Enhancement Induced by Ag/AgCl Nanoparticles

**Figure 7:**
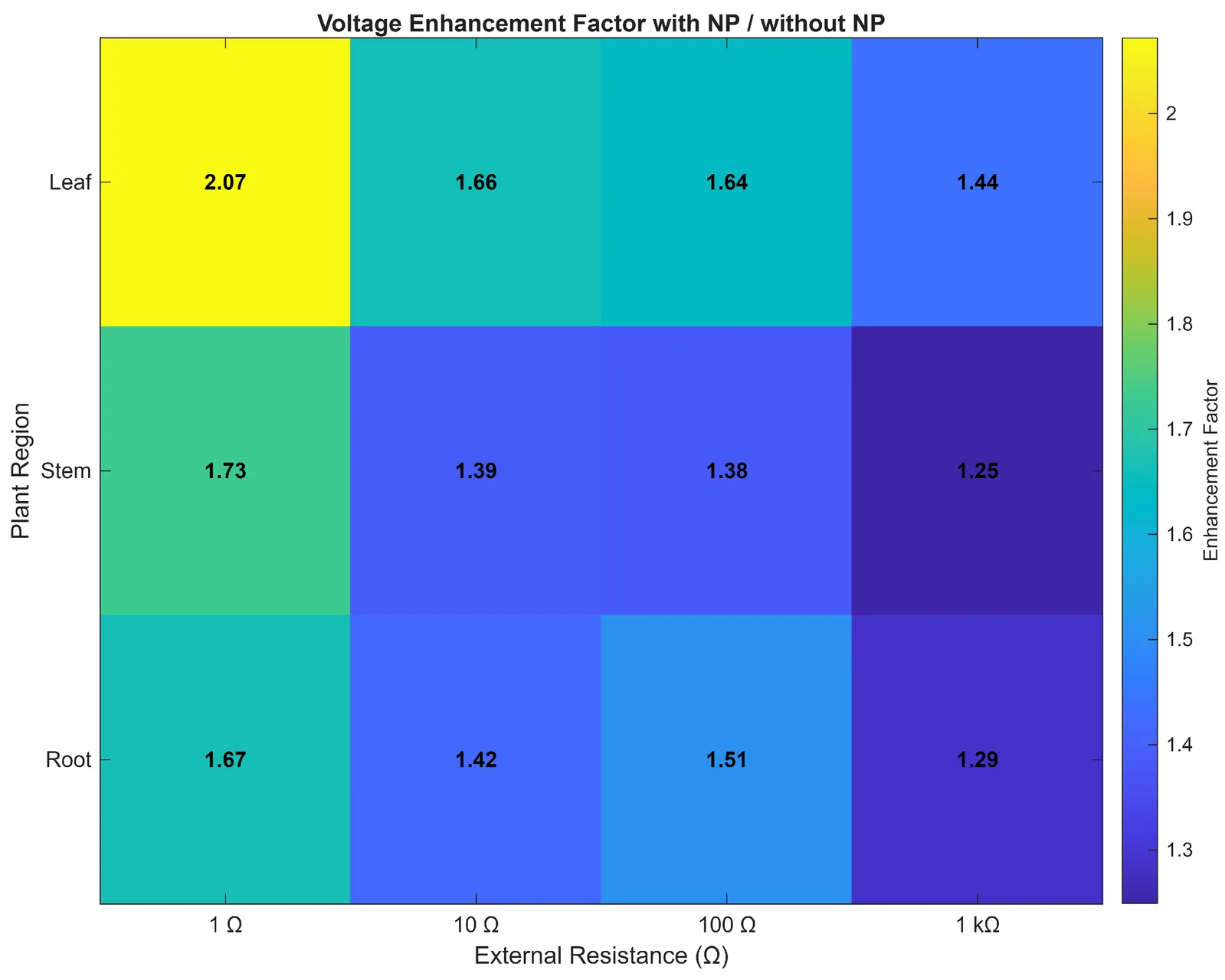
Voltage enhancement factor induced by Ag/AgCl nanoparticle incorporation across plant regions and external resistance conditions. The enhancement factor was calculated as the ratio between the complete voltage range ($V_{\max}-V_{\min}$) of the nanoparticle-incorporated plant and that of the control without nanoparticle incorporation over the same analyzed interval.

The voltage enhancement factor (VEF) was used to quantify changes in electrical signal amplitude between the Ag/AgCl NP-incorporated and control conditions. Figure 7 shows the VEF obtained for the evaluated tissues and external resistance conditions.

VEF values greater than 1 were obtained for all analyzed tissues and resistance conditions. The magnitude of the enhancement varied according to both plant tissue and external resistance. The largest enhancement was observed in leaf tissue at 1 Ω, where a VEF of 2.07 was obtained.

These measurements demonstrate that the magnitude of the electrical enhancement was dependent on both tissue type and external resistive load.

### 3.5. Thermal Sensitivity of Nanoparticle-Incorporated Plant Tissues

**Figure 8:**
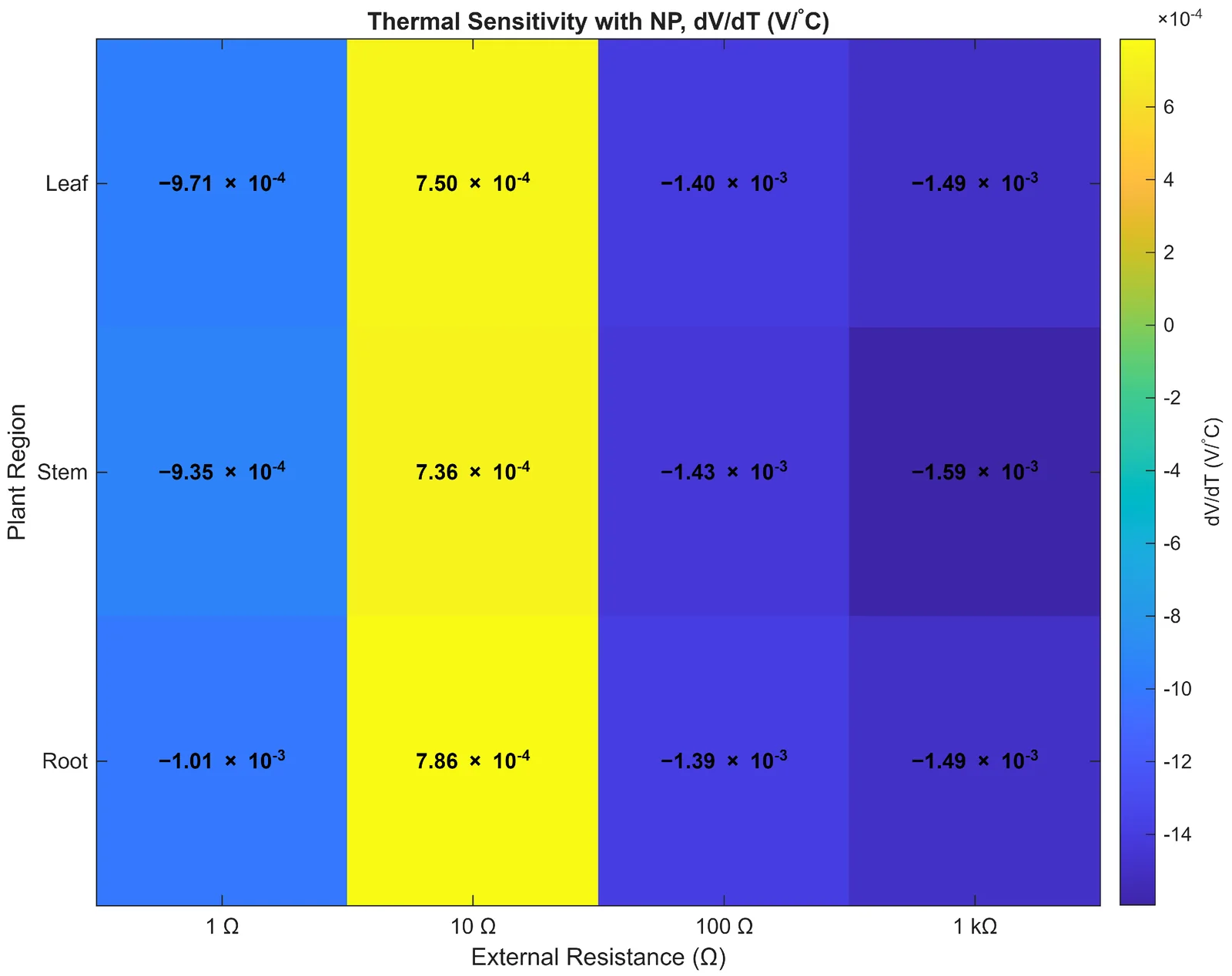
Voltage–temperature sensitivity of the Ag/AgCl NP-incorporated plant expressed as dV/dT (V °C^−1^) for leaf, stem, and root tissues under different external resistance conditions.

The voltage–temperature response of the Ag/AgCl NP-incorporated plant was quantified using dV/dT, expressed in V °C^−1^. The resulting values for leaf, stem, and root tissues under the evaluated external resistance conditions are summarized in Table 4 and visualized in Figure 8.

Positive values indicate an increase in voltage with increasing temperature, whereas negative values indicate the opposite voltage–temperature response.

Positive dV/dT values were observed only at 10 Ω for all three plant tissues, whereas negative values were obtained at 1, 100, and 1000 Ω. The highest positive value was observed in the root at 10 Ω ($7.86\times 10^{-4}$ V °C^−1^), while the most negative value was observed in the stem at 1000 Ω ($-1.59\times 10^{-3}$ V °C^−1^). Overall, both the magnitude and sign of dV/dT varied with plant tissue and external resistive load.

The positive dV/dT values at 10 Ω do not indicate that the measured voltages were positive. The voltage responses remained predominantly negative; however, the global linear fits indicated that the voltages tended to increase, or become less negative, as temperature increased. The reported coefficients represent the global voltage–temperature relationships over the complete analyzed interval rather than the local slopes around the small minima observed in the time-domain responses.

### 3.6. Voltage Fluctuation Dynamics and Electrothermal Coupling

**Figure 9:**
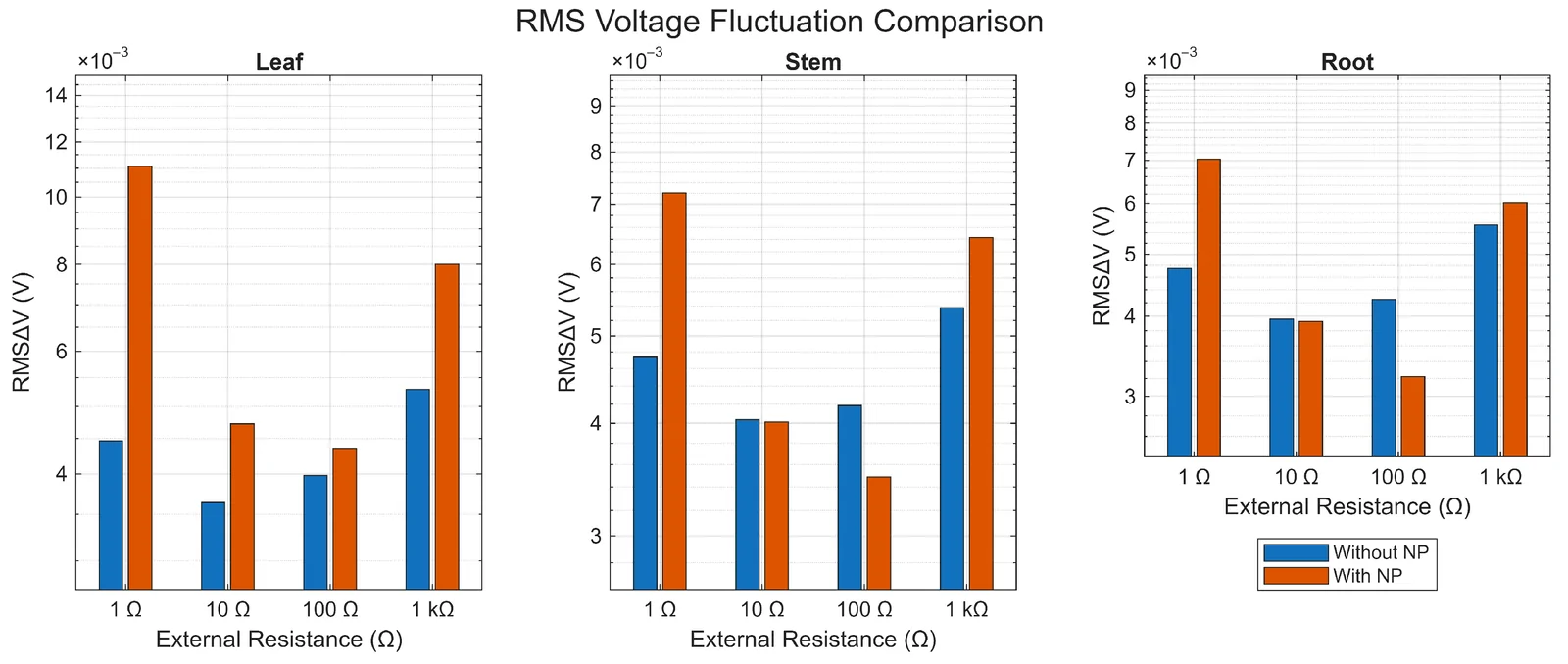
RMS voltage fluctuation (RMSΔ*V*) comparison between control and Ag/AgCl NP-incorporated *Epipremnum aureum* tissues under different external resistance conditions. RMSΔ*V* was calculated from first-order voltage differences and used as an indicator of short-term electrical signal variability.

Short-term electrical signal variations were quantified using the root mean square of the first-order voltage differences (RMSΔ*V*). Figure 9 compares RMSΔ*V* between the control and Ag/AgCl NP-incorporated conditions.

Differences between both experimental conditions were observed across the three plant tissues. Increased RMSΔ*V* values after nanoparticle incorporation were particularly evident under the 1 Ω and 1000 Ω conditions, whereas smaller or opposite changes were observed at the intermediate resistance values of 10 and 100 Ω. The magnitude of RMSΔ*V* therefore varied according to plant tissue and external resistance.

**Figure 10:**
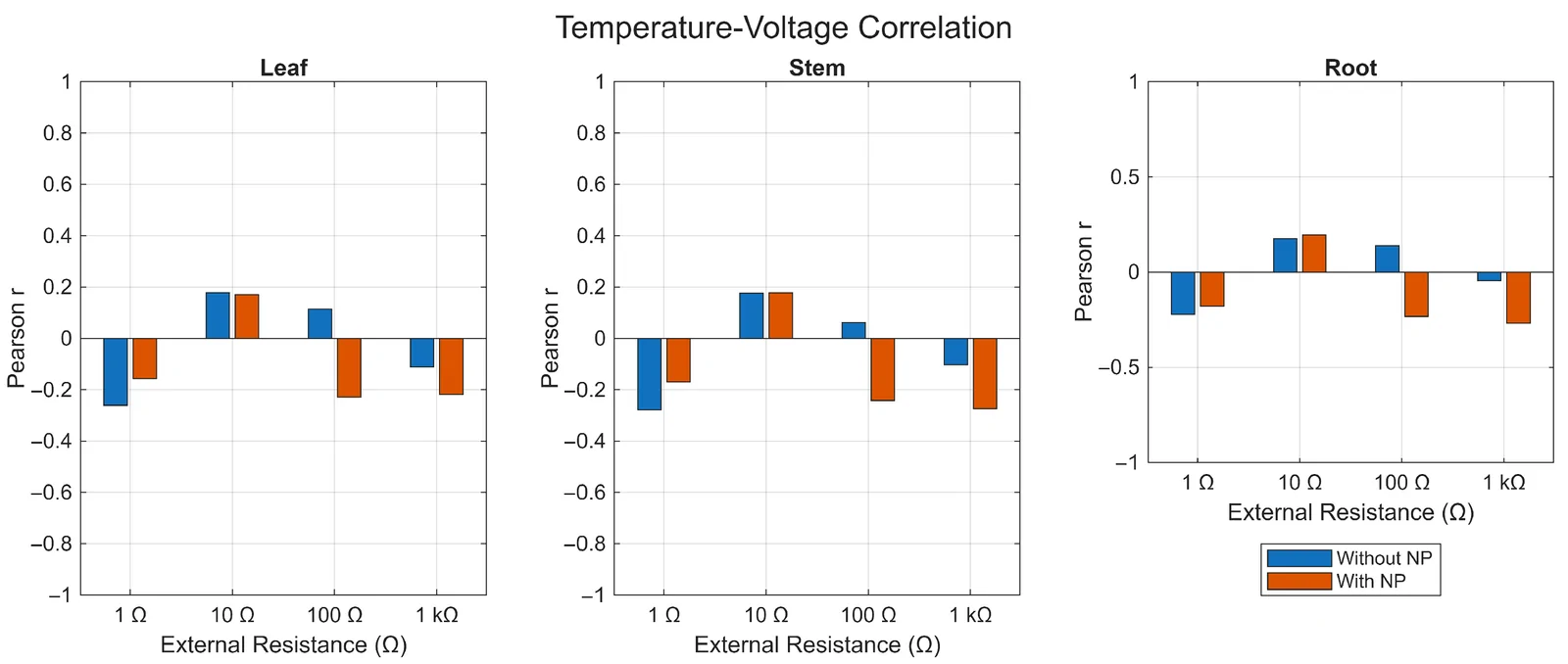
Temperature–voltage correlation analysis for control and Ag/AgCl NP-incorporated plant tissues. Pearson correlation coefficients were calculated for each tissue and external resistance condition.

Pearson correlation coefficients were calculated to quantify the linear association between plant temperature and voltage response. As shown in Figure 10, both the magnitude and sign of the correlation coefficients varied according to plant tissue, external resistance, and nanoparticle incorporation condition.

Positive correlations were observed under some intermediate resistance conditions, whereas negative correlations occurred under selected low- and high-resistance conditions. Overall, relatively low correlation coefficients were obtained, and a strong linear temperature–voltage relationship was not consistently observed across all evaluated conditions.

### 3.7. Morphological and Elemental Characterization of Nanoparticle-Incorporated Tissues

**Figure 11:**
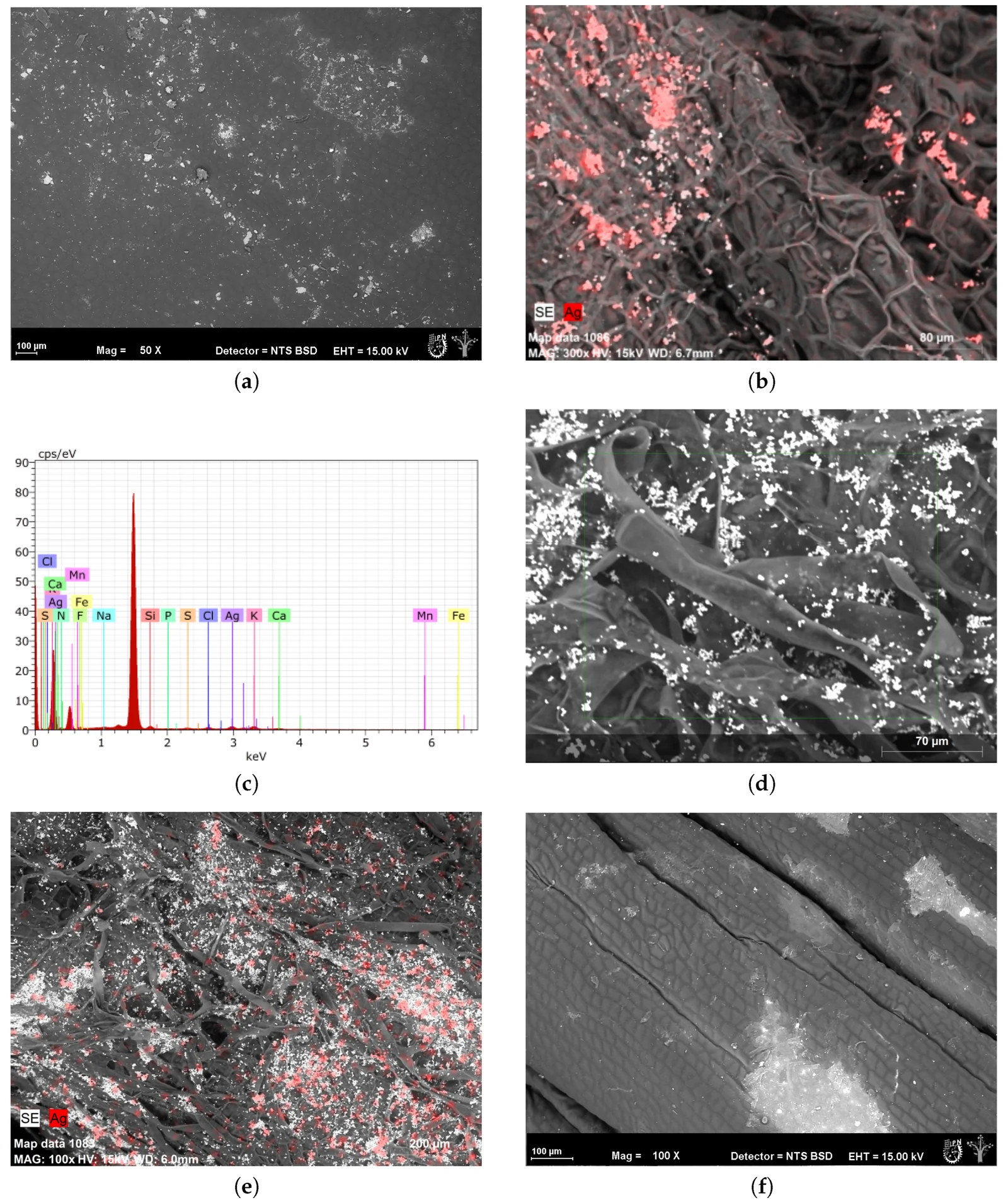
ESEM and EDX characterization of Ag/AgCl nanoparticle-incorporated *Epipremnum aureum* tissues. (**a**) ESEM micrograph of leaf tissue showing structural morphology. (**b**) EDX elemental mapping highlighting Ag distribution in leaf tissue. (**c**) Representative EDX spectrum showing Ag and Cl. (**d**) High-contrast root micrograph indicating Ag-rich domains; the green box indicates the region selected for EDX analysis. (**e**) EDX elemental mapping of stem tissue showing Ag distribution. (**f**) ESEM micrograph of stem tissue.

The presence and distribution of Ag-containing regions in *Epipremnum aureum* tissues were evaluated by ESEM and EDX. Representative micrographs, elemental maps, and an EDX spectrum are shown in Figure 11.

ESEM imaging showed heterogeneous bright regions within the analyzed plant tissues. EDX elemental mapping detected Ag-containing regions in the leaf and stem samples, while the representative EDX spectrum showed signals corresponding to Ag and Cl. Ag-rich regions were also identified in the root sample using compositional contrast.

Semiquantitative EDX analysis of two representative Ag-rich regions yielded Ag contents of 18.67 wt% and 14.34 wt%, respectively. Chlorine contents of 0.93 wt% and 0.78 wt% were measured in the same regions, as summarized in Table 5.

For reference, our previous EDX characterization of control *Epipremnum aureum* tissues from the same plant system reported normalized background chlorine contents of 0.72 wt% in the root, 1.50 wt% in the stem, and 0.54 wt% in the leaf [21]. These values indicate the presence of endogenous background chlorine in untreated plant tissues. Because these measurements were obtained from previously characterized samples and different regions of interest, they were not used for a direct quantitative comparison with the nanoparticle-incorporated tissues examined in the present study. Therefore, chlorine detection alone was not considered conclusive evidence of Ag/AgCl incorporation. Instead, the presence of Ag-rich regions, together with the simultaneous detection of Ag and Cl in the nanoparticle-incorporated tissues, supported the incorporation of the Ag/AgCl nanostructures.

### 3.8. Statistical Plant Tissue Region Comparison

A Wilcoxon signed-rank test was applied at a significance level of 5% (α=0.05) to evaluate the paired comparisons between the root and stem regions and between the stem and leaf regions illustrated in Figure 3. The analysis was performed for each of the evaluated external resistance conditions. The resulting *p*-values are summarized in Table 6.

For all evaluated resistance conditions, the obtained *p*-values were greater than 0.05. Therefore, no statistically significant differences were detected between the root and stem or between the stem and leaf voltage responses. The interpretation of these statistical findings in relation to the ESEM–EDX results and the electrical response across the evaluated plant tissue regions is presented in Section 4.

## 4. Discussion

The results obtained in this study support the potential of *Epipremnum aureum* incorporating Ag/AgCl nanostructures supported on nanocellulose as a living biohybrid sensing platform. Compared with the control condition without nanoparticle incorporation, nanoparticle incorporation was associated with changes in voltage amplitude, electrothermal sensitivity, and short-term signal dynamics. These observations are consistent with an interaction between the incorporated nanostructures and the electrical transport processes occurring within the plant tissues. However, given the proof-of-concept nature of the experimental design, these results should be interpreted as evidence of altered electrical behavior rather than as direct demonstration of a specific charge-transport mechanism.

The voltage enhancement factor (VEF) provided quantitative evidence of the change in electrical signal amplitude associated with Ag/AgCl incorporation. VEF values greater than unity were obtained under the evaluated tissue and resistance conditions, with the largest enhancement observed in leaf tissue at 1 Ω (VEF = 2.07). The dependence of VEF on both tissue type and external resistive load indicates that the electrical effect of nanoparticle incorporation was not uniform throughout the plant. This behavior is compatible with the complex electrical properties of living tissues, where ionic conduction, membrane polarization, tissue architecture, electrode interfaces, and external loading can jointly influence the measured voltage response [1,3,5].

The resistance-dependent behavior was also evident from the voltage–temperature sensitivity (dV/dT). Positive dV/dT values were observed at 10 Ω, whereas negative values were obtained at 1, 100, and 1000 Ω for all three evaluated tissues. Therefore, nanoparticle incorporation cannot be described simply as producing a uniform increase in voltage with increasing temperature. Instead, both the magnitude and direction of the electrothermal response depended on the electrical loading condition. This response is consistent with the nonlinear and non-ohmic behavior expected from a heterogeneous biological system in which ionic transport, membrane processes, interfacial effects, and externally imposed resistance contribute simultaneously to the measured electrical signal.

The root mean square of the first-order voltage differences (RMSΔ*V*) analysis further showed that Ag/AgCl incorporation affected the temporal dynamics of the electrical response. Increased RMSΔ*V* values were particularly evident at 1 and 1000 Ω, whereas smaller or opposite changes occurred at 10 and 100 Ω. Consequently, nanoparticle incorporation did not produce a uniform increase in short-term voltage fluctuations. Rather, its effect depended on the external resistive load and plant tissue. This finding suggests that the incorporated nanostructures may modify local electrical and electrochemical interactions within the biological matrix, although the present measurements do not allow the individual contributions of ionic transport, membrane responses, and nanoparticle-assisted conduction to be separated.

Plant electrical signaling is known to arise from coupled ionic, electrophysiological, hydraulic, and metabolic processes and can propagate through vascular tissues in response to environmental stimulation [1,2,4,5]. Moderate thermal stimulation has also been associated with propagating electrical signals and systemic physiological responses in higher plants [6,9,10]. In this context, the nonlinear responses, temporal asymmetry, and resistance-dependent modulation observed in the present study are compatible with a biological electrical system whose response is influenced by both intrinsic plant processes and the presence of Ag/AgCl-containing regions.

The Pearson correlation analysis provides additional evidence of this complexity. Relatively low and condition-dependent temperature–voltage correlation coefficients were obtained, including changes in correlation sign with tissue type, resistance, and nanoparticle incorporation condition. Therefore, temperature alone did not show a consistently strong linear relationship with the measured voltage response. This observation suggests that the electrical signals cannot be described by a simple linear electrothermal relationship and that additional physiological, electrochemical, and interfacial processes may contribute to the measured response. The coexistence of positive and negative dV/dT values under different resistive loads further supports this interpretation.

Structural and compositional characterization provides complementary evidence for interpreting these electrical changes. ESEM observations revealed localized bright regions, while EDX mapping showed a heterogeneous distribution of Ag-containing domains in the analyzed tissues. Rather than forming a continuous coating, the Ag-containing material appeared as localized enriched regions. Such heterogeneity could arise from preferential uptake, transport, retention, or deposition within particular anatomical regions of the plant. This behavior is relevant in the context of plant nanobionics, where vascular and tissue structures can influence nanomaterial transport and localization [12,13,14].

The simultaneous detection of Ag and Cl in the analyzed regions is consistent with the incorporation of the Ag/AgCl-containing material into the plant matrix. Semiquantitative EDX measurements yielded local Ag contents of 14.34–18.67 wt% in selected Ag-rich regions. These values represent localized measurements and should not be interpreted as the overall Ag concentration of the plant. Nevertheless, together with the EDX elemental maps, they confirm that silver-containing material was present in the tissues following the 12-week exposure period.

However, EDX provides semiquantitative elemental information and cannot determine chemical bonding, crystalline phase, or an exact Ag:Cl stoichiometric ratio. The comparatively lower Cl signal may be influenced by the heterogeneous distribution of the incorporated material, the native chlorine content of the plant tissues, the selected analysis area, and contributions from the surrounding biological matrix. Therefore, the co-detection of Ag and Cl should not be interpreted as conclusive evidence that all detected silver remained in the form of stoichiometric AgCl after incorporation into the plant. Conventional XRD analysis of the nanoparticle-incorporated tissues was not performed in the present study, and phase identification may be limited by the relatively low and heterogeneous nanostructure content and the complex organic plant matrix. Future studies should include direct comparisons with control tissues without nanoparticle incorporation and phase- or chemical-state- sensitive techniques, such as XPS, Raman spectroscopy, X-ray absorption spectroscopy, or spatially resolved micro-XRD, to determine the local chemical form of silver and its association with chlorine.

The coexistence of localized Ag-containing regions and changes in electrical behavior is consistent with the hypothesis that these nanostructures influence local charge-transport pathways or interfacial electrical processes within the plant. Conductive nanostructures incorporated into biological tissues may provide localized sites that affect ion and charge redistribution without requiring uniform coverage of the entire tissue. This interpretation is also consistent with our previous observations of silver-nanoparticle incorporation in *Epipremnum aureum*, where structural characterization and electrical measurements indicated nanoparticle-associated changes in plant electrical response [21]. The conceptual role proposed for these localized regions is illustrated schematically in Figure 1; however, the present ESEM–EDX and electrical measurements do not establish a direct continuous conductive pathway, and additional spatially resolved experiments would be required to confirm such a mechanism.

The statistical comparison between plant interfaces provides another relevant observation. The Wilcoxon signed-rank analysis yielded *p*-values greater than 0.05 for all evaluated resistance conditions, indicating that statistically significant differences were not detected between the Root–Stem and Stem–Leaf voltage responses under the conditions analyzed. Within the limitations of the present proof-of-concept dataset, this result indicates that the measured electrical response was not restricted to a single evaluated interface. Together with the detection of Ag-containing regions by ESEM–EDX, these findings provide supporting evidence that the incorporated Ag/AgCl nanoparticles reached different parts of the plant through its vascular system, contributing to the spatially distributed electrical response observed in the root, stem, and leaf regions.

From a sensing perspective, the results support viewing the nanoparticle-incorporated plant as an active biological transduction system whose electrical response contains information related to the applied thermal conditions. Plant bioelectrical signals have previously been shown to encode physiologically and environmentally relevant information that can be analyzed using data-driven approaches [7,8]. The incorporation of conductive nanostructures therefore provides a potential strategy for coupling plant electrophysiology with signal-processing and machine-learning methods in biohybrid sensing platforms.

This interpretation is supported by the regression analysis. Among the five evaluated models, MLP FitRNet achieved the best overall performance, with an MAE of 0.598 °C, an RMSE of 0.748 °C, and an $R^{2}$ value of 0.974 on the independent testing subset. Gradient Boosting produced comparable performance, and the remaining models also yielded high $R^{2}$ values. These results demonstrate that the acquired electrothermal voltage signals contained sufficient information for data-driven temperature estimation within the experimental dataset.

The high predictive performance of the regression models despite the relatively weak pairwise Pearson correlations is particularly noteworthy. Pearson correlation quantifies only linear association between individual voltage and temperature measurements under each condition, whereas machine-learning models can exploit more complex relationships present in the predictor space. Therefore, the two analyses are not necessarily contradictory. Instead, their combined results indicate that useful temperature-related information was present in the electrothermal signals even though the relationship was not consistently described by a simple linear correlation across tissues and resistance conditions. The slightly better performance of MLP FitRNet relative to the other models may reflect nonlinear components of this relationship; however, the similar performance among several regression approaches indicates that this interpretation should remain cautious.

The relatively small voltage changes observed above approximately 35 °C and the occurrence of similar voltage values at different temperatures further demonstrate that the electrothermal response is not a single-valued function of temperature. This behavior may arise from the combined and potentially competing effects of ion mobility, cellular membrane polarization within the plant tissues, tissue hydration, electrode–tissue interfacial polarization, and thermal equilibration within the plant. These processes can produce response plateaus, temporal delays, and hysteresis, such that similar instantaneous voltage values may occur during different stages of the thermal cycle or in different tissues. Therefore, voltage alone should not be interpreted as a direct and unique thermometric variable.

The regression models did not infer temperature from a single voltage value. Instead, they used the joint predictor space formed by the voltage responses of the control and Ag/AgCl NP-incorporated conditions. Nonlinear models can identify combinations and local relationships between these predictors that are not evident from individual voltage–temperature plots or pairwise linear correlations. Nevertheless, machine learning cannot unambiguously resolve cases in which exactly the same predictor combination corresponds to different temperatures. The reported performance should therefore be interpreted as interpolation within the experimentally sampled predictor space and temperature range, rather than as evidence of a unique physical voltage–temperature mapping or guaranteed extrapolation beyond the evaluated conditions. File-level partitioning reduced direct information leakage between training and testing subsets; however, temporal correlation within the continuous recordings remains a limitation and may contribute to the high predictive performance.

Plant nanobionics has demonstrated that engineered nanomaterials can augment or modify plant functions and enable new sensing capabilities [11,13,14]. The present results extend this concept to the investigation of electrothermal responses in Ag/AgCl nanoparticle-incorporated *Epipremnum aureum*. The convergence of VEF, dV/dT, RMSΔ*V*, temperature–voltage correlation, ESEM–EDX, and machine-learning results provides complementary evidence that nanoparticle incorporation is associated with measurable changes in the electrical behavior of the plant and that these signals can be computationally exploited for temperature estimation.

The present results should be interpreted within the scope of a proof-of-concept study. The electrothermal experiments were conducted using one Ag/AgCl NP-incorporated *Epipremnum aureum* specimen and one control without nanoparticle incorporation. The extensive dataset, comprising repeated measurements across multiple temperatures, tissue regions, and external resistance conditions, enabled a systematic evaluation of the electrothermal behavior and technical repeatability of the proposed platform.

Finally, the Wilcoxon signed-rank analysis was used to evaluate differences among tissue regions. Likewise, the machine-learning results demonstrate the feasibility of estimating temperature from the electrothermal signals under the experimental conditions represented in the dataset.

## 5. Conclusions

Ag/AgCl nanoparticle incorporation into *Epipremnum aureum* was associated with measurable, tissue- and resistance-dependent changes in the electrothermal response. The largest voltage enhancement was observed in leaf tissue at 1 Ω (VEF = 2.07). Variations in dV/dT, RMSΔ*V*, and Pearson correlation coefficients showed that the voltage–temperature relationship was nonlinear and dependent on the plant tissue and external electrical load. ESEM–EDX analysis identified localized Ag-containing regions, with Ag contents of 14.34–18.67 wt% in the selected Ag-rich areas. The Wilcoxon signed-rank test detected no statistically significant differences between the root and stem or between the stem and leaf regions under all evaluated resistance conditions. Together with the ESEM–EDX findings, these results provide supporting evidence that the incorporated Ag/AgCl nanoparticles reached different parts of the plant through its vascular system, contributing to the spatially distributed electrical response observed in the root, stem, and leaf regions.

Machine-learning analysis showed that the electrothermal signals contained sufficient information for temperature estimation within the experimental dataset. Among the evaluated models, MLP FitRNet achieved the best performance, with an MAE of 0.598 °C, an RMSE of 0.748 °C, and an $R^{2}$ of 0.974. The proposed method demonstrated that incorporating Ag/AgCl nanoparticles into *Epipremnum aureum* amplifies the electrothermal signal; however, certain limitations remain, revealing opportunities for future work, such as extending the study to other plant species, analyzing the stability and durability of Ag/AgCl nanoparticles within the tissues over prolonged periods, and investigating the influence of humidity, lighting, irrigation, pH, and nutrient availability. Furthermore, future studies should compare additional AI architectures and develop models that integrate voltage, temperature, humidity, and other environmental variables to detect water stress, nutritional deficiencies, and other physiological states. 

## Figures and Tables

**Table 1:** Variables used for machine-learning-based temperature prediction.

Variable	Description	Role
WithoutNP_Voltage_V	Voltage measured in the plant without nanoparticle incorporation	Predictor
WithNP_Voltage_V	Voltage measured in the Ag/AgCl NP-incorporated plant	Predictor
Temperature_C	Measured plant temperature	Target

**Table 2:** Distribution of acquisition files and observations among the training, validation, and independent testing subsets.

Subset	Files	Observations	Percentage
Training	238	47,600	70%
Validation	51	10,200	15%
Testing	51	10,200	15%

**Table 3:** Performance of machine-learning regression models for temperature estimation from electrothermal voltage signals.

Model	MAE (°C)	RMSE (°C)	MBE (°C)	*R* ^2^	Max Abs Error (°C)
MLP FitRNet	0.598	0.748	0.036	0.974	2.766
Gradient Boosting	0.601	0.752	0.034	0.974	2.930
SVR Gaussian	0.636	0.762	0.036	0.973	2.645
Linear Regression	0.613	0.763	0.066	0.973	2.807
Random Forest	0.648	0.818	0.034	0.969	3.199

**Table 4:** Voltage–temperature sensitivity (dV/dT) of Ag/AgCl NP-incorporated *Epipremnum aureum* tissues under different external resistance conditions.

Resistance	Leaf	Stem *dV*/*dT* (V °C^−1^)	Root
1 Ω	$-9.71\times 10^{-4}$	$-9.35\times 10^{-4}$	$-1.01\times 10^{-3}$
10 Ω	$7.50\times 10^{-4}$	$7.36\times 10^{-4}$	$7.86\times 10^{-4}$
100 Ω	$-1.40\times 10^{-3}$	$-1.43\times 10^{-3}$	$-1.39\times 10^{-3}$
1000 Ω	$-1.49\times 10^{-3}$	$-1.59\times 10^{-3}$	$-1.49\times 10^{-3}$

**Table 5:** Semiquantitative EDX composition of two representative nanoparticle-rich regions identified in ESEM micrographs of nanoparticle-incorporated *Epipremnum aureum* tissues.

Element	Region 1 (wt%)	Region 2 (wt%)
Silver (Ag)	18.67	14.34
Chlorine (Cl)	0.93	0.78
Sulfur (S)	0.50	0.60
Sodium (Na)	–	1.05
Silicon (Si)	0.65	0.63
Magnesium (Mg)	0.95	0.98
Calcium (Ca)	–	–

**Table 6:** Results of the Wilcoxon signed-rank test for the paired comparisons between plant tissue regions under different external resistance conditions. The significance level was set at 5%.

Electrical Resistance	*p*-Value		Remarks
	Root–Stem	Stem–Leaf	
1 Ω	$4.1870\times 10^{-1}$	$2.6915\times 10^{-1}$	No statistically significant differences were detected in either comparison.
10 Ω	$5.2183\times 10^{-1}$	$5.3721\times 10^{-1}$	No statistically significant differences were detected in either comparison.
100 Ω	$4.2913\times 10^{-1}$	$2.5245\times 10^{-1}$	No statistically significant differences were detected in either comparison.
1000 Ω	$4.8566\times 10^{-1}$	$2.0069\times 10^{-1}$	No statistically significant differences were detected in either comparison.

## Data Availability

The data presented in this study are available from the corresponding authors upon reasonable request.

## Abbreviations

Ag/AgCl	Silver/silver chloride
DAQ	Data acquisition system
EDX	Energy-dispersive X-ray spectroscopy
ESEM	Environmental scanning electron microscopy
MAE	Mean absolute error
MBE	Mean bias error
MLP	Multilayer perceptron
NP	Nanoparticle
RMS	Root mean square
RMSE	Root mean square error
$R^{2}$	Coefficient of determination
SVR	Support vector regression
wt%	Weight percentage
